# Comparison of CRISPR/Cas9 and TALENs on editing an integrated EGFP gene in the genome of HEK293FT cells

**DOI:** 10.1186/s40064-016-2536-3

**Published:** 2016-06-21

**Authors:** Zuyong He, Chris Proudfoot, C. Bruce A. Whitelaw, Simon G. Lillico

**Affiliations:** State Key Laboratory of Biocontrol, School of Life Sciences, Sun Yat-sen University, Guangzhou, 510006 People’s Republic of China; The Roslin Institute and Royal (Dick) School of Veterinary Studies, University of Edinburgh, Easter Bush Campus, Edinburgh, EH25 9RG UK

**Keywords:** CRISPR/Cas9, Deletion, HDR, TALEN

## Abstract

**Background:**

Genome editors such as CRISPR/Cas9 and TALENs are at the forefront of research into methodologies for targeted modification of the mammalian genome. To date few comparative studies have been carried out to investigate the difference of genome editing characteristics between CRISPR/Cas9 and TALENs. While the CRISPR/Cas9 system has overtaken TALENs as the tool of choice for most research groups working in this field, we hypothesized that there could be certain applications whereby the application of TALENs would have specific benefits. Here we compare CRISPR/Cas9 and TALEN as tools for introducing site-specific editing events at an integrated EGFP gene in the genome of HEK293FT cells.

**Results:**

Guide RNAs and TALEN pairs were designed to target two loci within the EGFP gene. We found that paired Cas9 nucleases induced targeted genomic deletion more efficiently and precisely than two TALEN pairs. However, when concurrently supplied with a plasmid template spanning the two DNA double-strand breaks (DSBs) within EGFP, TALENs stimulated homology directed repair (HDR) more efficiently than CRISPR/Cas9 and caused fewer targeted genomic deletions.

**Conclusions:**

Our data suggest that the choice of genome editing tool should be determined by the desired genome editing outcome. Such a rational approach is likely to benefit research outputs for groups working in fields as diverse as modification of cell lines, to animal models for disease studies, or gene therapy strategies.

**Electronic supplementary material:**

The online version of this article (doi:10.1186/s40064-016-2536-3) contains supplementary material, which is available to authorized users.

## Background

Until recently the precise modification of endogenous mammalian DNA sequences was not a practical goal for most research labs. The advent of genome editing tools has dramatically changed this landscape, with diverse groups around the world having embraced these new technologies. The CRISPR/Cas9 system in particular is being developed into a robust and multiplexable genome editing tool, enabling researchers to precisely manipulate specific genomic elements (Wiles et al. 2015). This system relies on the bacterial endonuclease Cas9, which can be guided by means of simple base-pair complementarity between the first 20 nucleotides of an engineered guide RNA (gRNA) and a target genomic DNA sequence of interest that lies next to a protospacer adjacent motif (PAM). The recruitment of Cas9 nuclease to the target site results in the generation of a DNA double-strand break (DSB). Subsequent error prone cellular DNA repair processes lead to the formation of insertions, deletions or substitutions at the target site (Jinek et al. 2012). CRISPR/Cas9 has been used to modify the genomes of human and murine cell lines with efficiencies comparable to those of established nucleases used for targeted genome engineering (Wiles et al. 2015) and now this approach has been extended to edit the genomes of model organisms, including zebrafish, mouse, rat, and pig (Ledford 2015).

Before the emergence of CRSISPR/Cas9, two protein-guided genome engineering systems, zinc-finger nucleases (ZFNs) and transcription activator like effector nucleases (TALENs), were widely used to modify endogenous genes in a wide range of cell lines and living organisms (Joung and Sander 2013). Using these technologies numerous genomic alterations have been reported, including insertions, deletions, point mutations, inversions, duplications and translocations, thus providing researchers with unprecedented tools to perform genetic manipulations. Compared with ZFNs, TALENs can be very easily and rapidly constructed using a simple “one-to-one” correspondence between single DNA base pairs in a target site and two-amino-acid sequences in one TAL effector repeat (Boch and Bonas 2010). A large-scale test demonstrated that TALENs have a very high success rate and can be used to target almost any DNA sequence of interest in human cells, enabling application as a broadly applicable genome editing tool (Reyon et al. 2012).

Both CRISPR/Cas9 and TALENs enable genomic engineering. TALENs use the FokI nuclease domain to induce site-specific DNA cleavage, and generate overhangs (Kim et al. 2013b). In contrast, in the CRISPR/Cas9 system, the Cas9 nuclease has been shown to cleave at a site three or four base pairs upstream of the PAM, and generate blunt ends (Jinek et al. 2012). Due to their different cleavage characters, we propose that these editors may be associated with different mutation patterns. HEK293 cell lines have been extensively used for testing for genome editing signatures of both TALENs and CRISPR/Cas9 (Kim et al. 2013a; Ren et al. 2015), therefore in this study we generated a HEK293FT cell line with a single copy insertion of enhanced green fluorescent protein (EGFP). EGFP can be converted to the closely related enhanced blue fluorescent protein(EBFP) through two substitutions, Y66H (199T > C) and Y145F (437A > T), where the Y66H substitution alters the chromophore π-electron structure within the EGFP which is critical for the blue-shifting, while the Y145F substitution improves the brightness and photostability of the EBFP (Pakhomov and Martynov 2008). TALENs and CRISPR/Cas9 were designed to target the two substitution sites within EGFP and a comparison of these editors was conducted to assess their efficiency as mediators of disruption or deletion within EGFP, or conversion to EBFP when utilized in conjunction with a template for HDR.

## Methods

### Editor design and construction

**Fig. 1:**
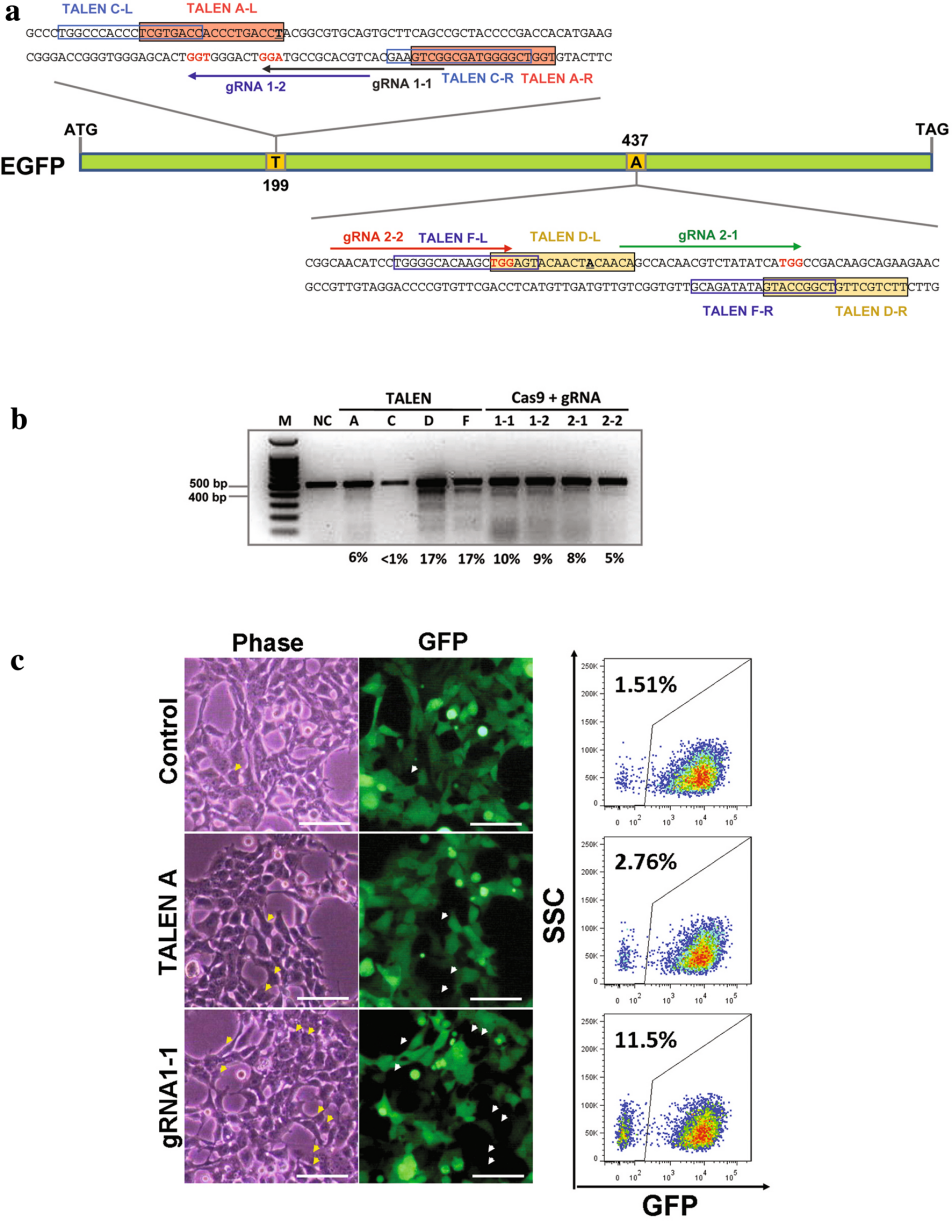
TALEN and gRNA design, and comparison of these gene editors on EGFP gene disruption. **a** Schematic diagram of target sites for TALENs and gRNAs proximal to the two substitution sites (199T > C) and (437A > T) for conversion of EGFP to EBFP. *Coloured arrow lines* indicate the sequence used for the guide segment of gRNAs. The NGG nucleotide protospacer adjacent motif (PAM) sequences in *red*. *Colour boxed* sequences denote the DNA binding regions of the TALEN proteins. **b** Surveyor nuclease assay of genomic DNA isolated from HEK293FT_EGFP_ cells with one copy of integrated EGFP gene expressing Cas9 and gRNAs, or TALENs. NC: genomic DNA from untransfected HEK293FT_EGFP_ cells was used a negative control. **c** The representatives of examination of EGFP negative cells in HEK293FT_EGFP_ cells transfected with Cas9 and gRNAs, or TALENs under a phase-contrast and fluorescence microscope, and quantification of EGFP negative cells using by fluorescence-activated cell sorter (FACS). *Arrow heads* indicate EGFP negative cells. *Scale bar* of 50 μm. For FACS analysis, in each case, a total of 10,000 events were counted

TALENs and gRNAs were designed to target the two substitution sites required to convert EGFP to EBFP. For each substitution site, two TALEN pairs and two gRNAs were designed using the ZiFiT Targeter software (Sander et al. 2010). TALENs were assembled using methods described previously (Carlson et al. 2012; Lillico et al. 2013). Briefly, intermediary arrays were produced for Golden Gate cloning as follows; 150 ng each pFUS_A, pFUS_B, pLR-X and pC- + 63-TAL modified vector were incubated for 10 cycles of 5 min at 37 °C and 10 min at 16 °C, then heated to 50 °C for 5 min and then 80 °C for 5 min in the presence of 50 units T4 DNA ligase (New England Biolabs), 10 units Esp3I (Fermentas), 1 × T4 ligase buffer (New England Biolabs) (TALEN RVD sequences in Additional file 6: Table 1). The plasmid encoding human codon optimized Cas9 gene (hCas9, plasmid number 41815, Addgene) was generated as described previously (Mali et al. 2013). Similar to our previous study (He et al. 2015), the target gRNA expression constructs were ordered as individual 455 bp gBlocks (IDT) (Sequence in Additional file 1: Figure  1A). Each gBlock was cloned into pUC19. The four gRNAs used in this study are listed in Additional file 1: Figure  1B. EBFP donor template and EBFP blocking mutation donor template were synthesized as gBlocks (IDT) (Additional file: 1 Figure  1C), and cloned into pUC19 using appropriate restriction enzymes. All oligonucleotide templates (Additional file 1: Figure  1D) were synthesized by IDT, 100 nmole synthesis purified by standard desalting, and resuspended to 400 μM in TE.

### Generation of HEK293FT_EGFP_ cell line using lentivirus

Human embryonic kidney 293FT cells were maintained in Dulbecco’s modified Eagle medium (Sigma) supplemented with 10 % fetal bovine serum. Cells were seeded at 3 × 10^5^ cells/well in a 12-well plate, cultured for 24 h, then transduced with a lentiviral vector containing CMV-EGFP at an MOI of 0.001. Following culture for a further 48 h, FACS was used to individually seed EGFP positive cells into wells of a 96-well plate. Clonal populations were expanded and all subsequent analysis was with a single clonal population of HEK293FT_EGFP_ cells shown by Southern blot to possess a single lentiviral insertion of the EGFP transgene (Additional file 2: Figure  2).

### Cell culture and transfection

HEK293FT_EGFP_ cells were seeded at 1.5 × 10^5^ cells/well in a 24-well plate, incubated for 24 h, and then transfected with 0.3 μg Cas9 plasmid and 0.1 μg of individual gRNA plasmid of a pair, using X-tremeGENE HP DNA transfection reagent (Roche). For TALENs, 0.1 μg of each plasmid of the TALEN pair were co-transfected using the same regime as above. At day 3 post-transfection, genomic DNA was isolated from cells using the DNeasy Blood & Tissue Kit (Qiagen), following the manufacturer’s protocol, and used for PCR analysis and Surveyor nuclease assay. For homologous recombination using plasmid templates, 0.2 μg Cas9 plasmid, 0.1 μg of individual gRNA plasmid of a pair and 0.1 μg donor plasmid were transfected into HEK293FT_EGFP_ cells; 0.1 μg of each plasmid of the TALEN pair and 0.1 μg donor plasmid were transfected into HEK293FT_EGFP_ cells. For homologous recombination using oligonucleotide templates, 0.2 μg Cas9 plasmid, 0.1 μg of individual gRNA plasmids and 0.05 μg single-stranded donor oligonucleotides (ssODN) were transfected; 0.1 μg of each plasmid of the TALEN pair and 0.05 μg ssODN were transfected into HEK293FT_EGFP_ cells.

### Flow cytometry

The fluorescence of EGFP and EBFP were detected by a FACSAria™ cytometer (BD Biosciences) equipped with a 488 nm argon laser and a 380-nm UV laser. Briefly, 5 × 10^5^ cells were harvested 7 days after transfection and resuspended in 1 ml DMEM medium supplemented with 5 % FBS. Cell debris was excluded from analysis using bivariate, forward/side scatter (FSC/SSC) parameters and dead cells were gated from analysis using propidium iodide (PI). 1 × 10^4^ gated events were acquired per sample and the values were calculated as a percentage of the cell population using FlowJo software (TreeStar, USA).

### SURVEYOR nuclease assay and PCR analysis of genome modification

HEK293FT_EGFP_ cells were transfected with DNA as described above. Cells were cultured for 3 days post transfection prior to genomic DNA extraction. Genomic DNA was purified using the DNeasy Blood & Tissue Kit (QIAGEN) following the manufacturer’s protocol. The genomic region flanking TALEN or gRNA target sites was PCR amplified using DreamTaq DNA polymerase (Thermo Scientific) and appropriate primers (Additional file 7: Table 2). SURVEYOR nuclease assay was carried out using methods described previously (Hsu et al. 2013). Briefly, 400 ng total of the PCR products were subjected to a denature/anneal process to enable heteroduplex formation: 95 °C for 10 min; 95–85 °C ramping at −2 °C/s; 85–25 °C at −0.25 °C/s; and 25 °C hold for 1 min. After reannealing, products were treated with SURVEYOR nuclease and SURVEYOR enhancer S (Transgenomics) following the manufacturer’s recommended protocol, and analyzed on 2 % agarose gels. Gels were imaged with a Gel Doc gel imaging system (Bio-Rad). Quantification was based on relative band intensities (Ran et al. 2013). Similar to our previous study (He et al. 2015), to detect genomic deletions and homologous recombination, genomic DNA was subjected to PCR analysis using DreamTaq DNA polymerase (Thermo Scientific) and appropriate primers (Additional file 7: Table 2). PCR products were analyzed by agarose gel electrophoresis. For sequencing analysis, PCR products corresponding to genomic deletions or homologous recombination were purified using a QIAquick Gel Extraction Kit (QIAGEN) and cloned into pGEM-T Easy vector (Promega). Cloned plasmids were sequenced using M13 primers.

### Statistical analysis

The counting of EGFP negative cells using FACS was performed in duplicate in three independent experiments, with the experimental data analyzed by one-way ANOVA using SPSS15.0 software (SPSS Inc., USA). The data are shown as the mean ± SE for three independent experiments.

## Results

### EGFP disruption with CRISPR/Cas9 or TALEN

To compare the gene editing ability of CRISPR/Cas9 and TALEN, we designed gRNAs and TALENs that targeted the two reported substitution sites on EGFP, Y66H (199T > C) and Y145F (437A > T), predicted to convert EGFP into EBFP (Pakhomov and Martynov 2008). First, gRNA 1-1, gRNA 1-2, TALEN pair A and TALEN pair C were designed to cleave DNA proximal to 199T. Similarly, gRNAs 2-1 and 2-2, and TALEN pairs D and F were designed to cleave DNA proximal to 437A (Fig. 1a). Note that gRNA 1-1 and gRNA 1-2 have a 14 nt overlap and that they target the opposite strand to gRNA 2-1 and gRNA 2-2. The binding sites of TALEN pairs A and C both target the same overlapping region, with TALEN pair C having a longer spacer than pair A. This also applies to TALEN pairs D and F.

Each CRISPR/Cas9 or TALEN pair was transfected into HEK293FT_EGFP_ cells, and the efficiency of genome modification at day 3 was measured by Surveyor assay. Each of the four gRNAs induced NHEJ at their respective target sites, with activity ranging from 5 to 10 % (Fig. 1b). TALEN pair A displayed a cleavage activity comparable to gRNA 2-2 while TALEN pair C had a cleavage activity below the detection limit of the Surveyor assay. TALEN pair D and F displayed the most active cleavage activity of 17 %. Seven days post-transfection, fluorescence microscopy showed more EGFP negative cells following treatment with CRISPR/Cas9 or TALEN than in the control population (we observed a consistent low background of EGFP negative cells in our HEK293FT_EGFP_ population despite re-isolation by FACS), confirmed by FACS analysis (Fig. 1d).

### Paired CRISPR/Cas9 is both more efficient and precise at generating deletions than paired TALEN pairs

**Fig. 2:**
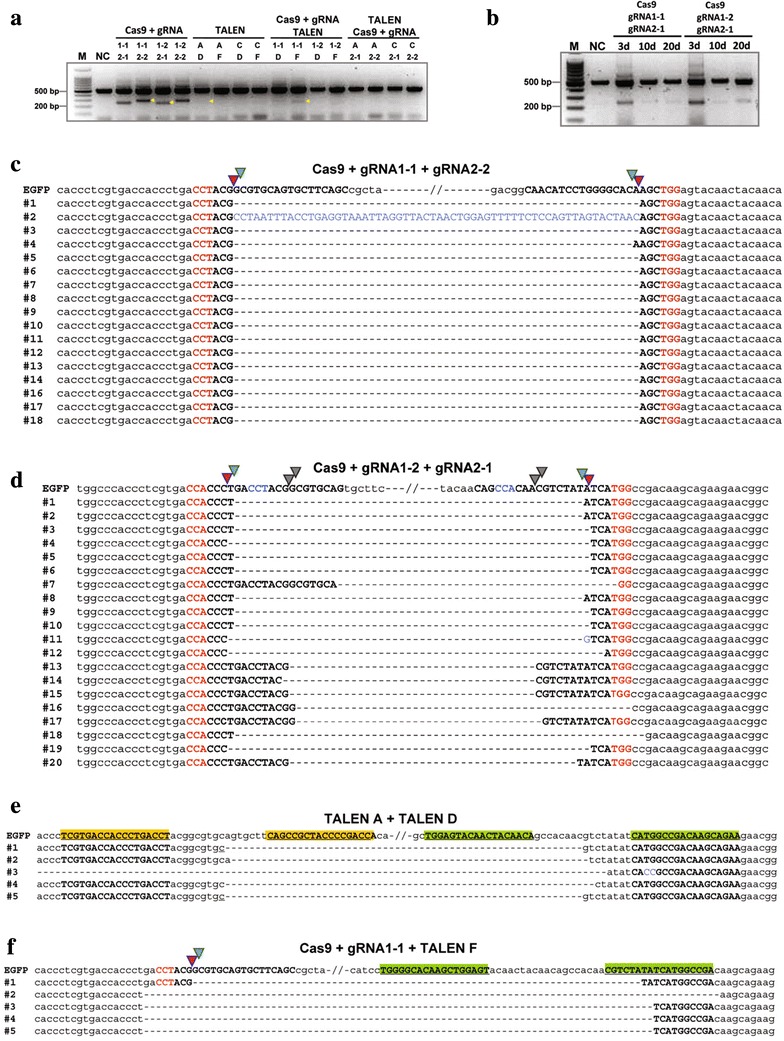
A comparison of TALEN and CRIPSR/Cas9 on inducing targeted genomic deletions. **a** Genomic deletions detected using PCR. *Arrow heads* indicate deletion-specific PCR products cloned for sequencing. **b** Genomic deletions detected at day 3, day 10 and day 20 after HEK293FT_EGFP_ cells transfected with paired Cas9. **c** DNA sequences of deletion-specific PCR products obtained using gRNA 1-1, gRNA 2-2 and Cas9. Target site PAM sequences in *red*, and gRNA-matching sequences in *bold upper case letters*. Predicted cleavage sites represented by *green* and *red arrow heads*. *Inserted* bases in *blue*. *Dashes* indicate deleted bases. **d** DNA sequences of deletion-specific PCR products obtained using gRNA 1-2, gRNA 2-1 and Cas9. The suspected second PAM in gRNAs is shown in *blue*, and predicted cleavage sites around second PAM are represented by *grey arrow heads*. **e** DNA sequences of deletion-specific PCR products obtained using TALEN pair A and D. The target sites of TALEN pair A in *underlined boldface letters*, and highlighted in *yellow*. The target sites of TALEN pair D are highlighted in *green*. Microhomologies *underlined*. **f** DNA sequences of deletion-specific PCR products obtained using gRNA 1-1, Cas9 and TALEN pair F

To compare the ability to delete genomic regions, HEK293FT_EGFP_ cells were cotransfected with plasmids encoding Cas9 and a pair of gRNAs, two pairs of TALENs or a pair of TALENs combined with one CRISPR/Cas9. Three days post transfection, genomic DNA was prepared from cell pools and analysed by PCR. PCR across the target region using primers site1 F and site2 R (Additional file 7: Table 2) produced a full length product of 509 bp, or in the event of a genomic deletion between target sites a product approximating 250 bp. The truncated PCR product was most apparent in cells transfected with paired CRISPR/Cas9 nuclease reagents, less apparent when gRNA 1-1 was paired with TALEN pair D or F, and barely observed when TALEN pair A was combined with pair D (Fig. 2a). Following extended culture the deletion frequency declined by day 10 and thereafter remained stable until day 20 (Fig. 2b), consistent with data previously reported for NHEJ-induced indels (Carlson et al. 2012).

Deletion-specific PCR products (yellow arrow heads Fig. 2a) were cloned and sequenced, confirming that in each case the intervening DNA segment had been deleted (Fig. 2c–f). Truncated DNA generated through the pairing of gRNA 1-1 and gRNA 2-2 revealed that the majority of events (16/17) represented a precise ligation ofthe breakpoint junctions generated by Cas9 (Fig. 2c).

ZFNs and TALENs both use a fused FokI nuclease to cleave their double-stranded DNA target sites, generating heterogeneous ends which are then rejoined through NHEJ. The resultant breakpoint junction sequences often show small indels associated with micro-homologies of 1–5 bases (Lee et al. 2010; Xiao et al. 2013). From five sequenced clones derived from TALEN pair A and D, we observed deletion and micro-homology repair at the breakpoint (Fig. 2e). When genomic deletion was generated by the combination of CRISPR/Cas9 and a TALEN pair, additional deletions were more often observed on both breakpoint junctions (Fig. 2f). It is known that the NHEJ ligation step can be preceded by annealing over micro-homology of a few base pairs, leading to potential deletion of unpaired bases (Ray and Langer 2002). Therefore, it is possible that the overhangs generated by the FokI of TALEN pair F and not the Cas9 nuclease was largely responsible for the observed deletions that were induced during the ligation process.

### Two TALEN pairs mediate HDR more efficiently than paired gRNAs

**Fig. 3:**
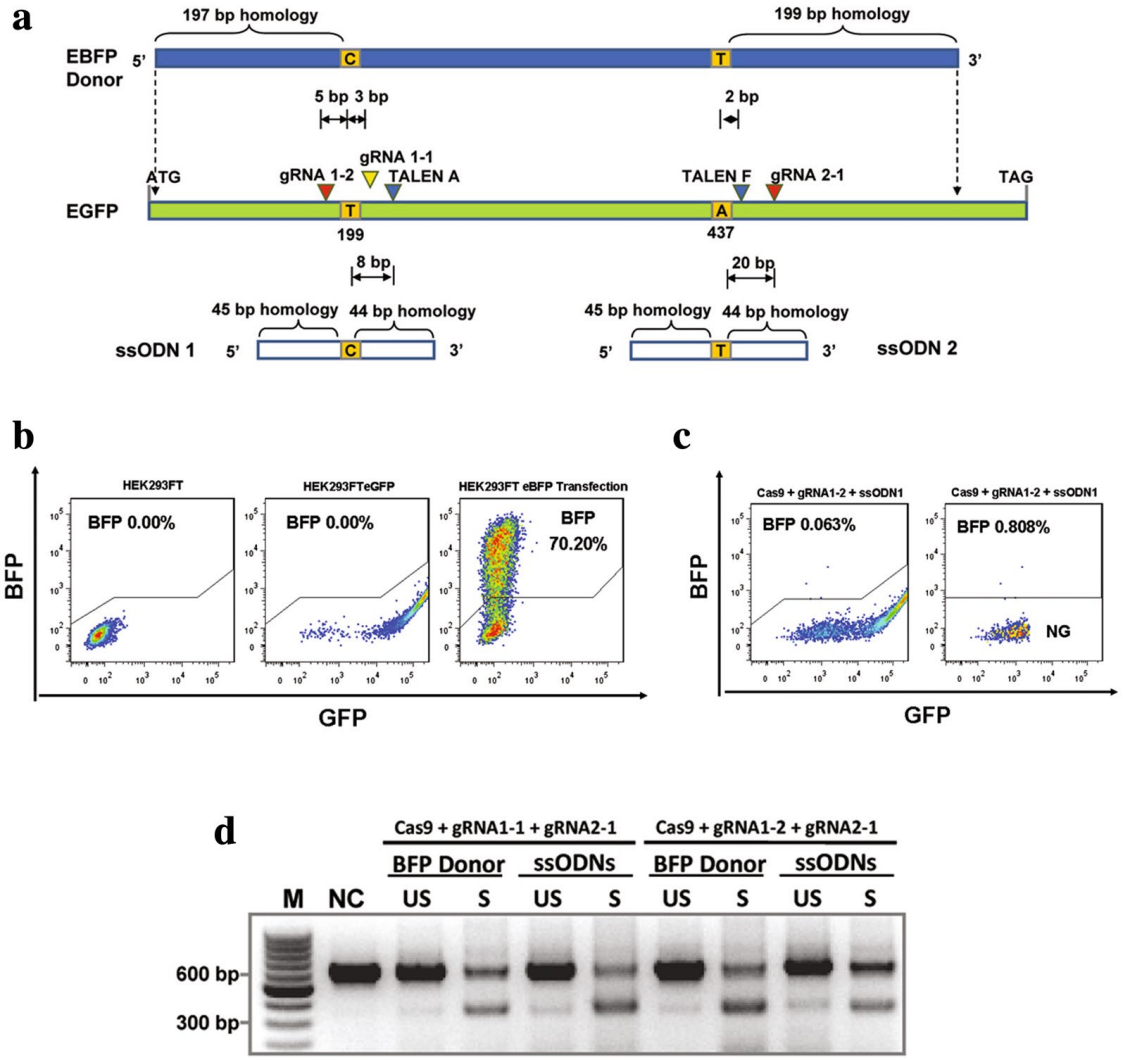
The design of single-stranded oligodeoxynucleotides (ssODNs) and plasmid donor, and the comparison of HDR efficiencies between TALEN and CRISPR/Cas9. **a** The schematic diagram shows two 90-mer ssODN and a plasmid donor DNA used to incorporate the two substitutions (199T > C) and (437A > T) into the EGFP locus. Predicted cleavage sites of CRISPR/Cas9 or TALEN are indicated by *coloured arrow heads*. **b** The set up of controls for FACS analysis. HEK293FT cell line was used as EGFP and EBFP negative control. HEK293FT_EGFP_ cell line was used as EGFP positive and EBFP negative control. HEK293FT cells transiently transfected with a plasmid encoding EBFP were used as EBFP positive control. In each case, a total of 10,000 events were counted. **c** Representatives of the FACS analysis of EBFP positive cells generated via HDR by using TALEN or CRISPR/Cas9 with donor templates. NG: EGFP negative cells. **d** Detection of targeted genomic deletions in sorted EGFP negative populations using PCR. NC: genomic DNA from untransfected HEK293FT_EGFP_ cells was used as negative control. US: genomic DNA from HEK293FT_EGFP_ cells transfect paired Cas9 and ssODN or plasmid donor was used for PCR. S: genomic DNA from sorted EGFP negative cells from HEK293FT_EGFP_ cells transfected with paired Cas9 and ssODN or plasmid donor

Previous reports on TALEN or CRISPR/Cas9 mediated homology-directed repair (HDR) using plasmid or ssODN templates have focused on conversion of a single site. Here we compared CRISPR/Cas9 and TALEN for the simultaneous substitution of 2 bases within the EGFP gene separated by 238 bp, with the aim of converting EGFP to EBFP (Fig. 3a). Co-transfection of gRNA 1-2, Cas9 andssODN1 resulted in approximately 0.06 % EBFP positive cells, with this number rising to 0.8 % EBFP positive cells within the EGFP negative population (Fig. 3b, c; Additional file 8: Table 3). Concurrent delivery of 2 guides (gRNA 1-2 and gRNA 2-1) and 2 templates (ssODN1 and ssODN2) produced a comparable proportion of EGFP negative cells as compared to delivery of a single guide (8.21 vs 8 %), with less than 0.01 % of these cells being EBFP positive (Additional file 3: Figure  3A, Additional file 8: Table 3). This suggests that concurrent DSBs induced by the paired gRNA/Cas9 resulted in preferential deletion of the intervening DNA rather than supporting HDR. This is corroborated by the increased frequency of the truncated PCR product in the FACS sorted EGFP negative cells relative to the unsorted population (Fig. 3d). When two TALEN pairs (A and F) together with the same ssODN1 and ssODN2 were introduced into HEK293FT_EGFP_ cells, FACS analysis revealed that 0.035 % became EBFP positive (1.92 % of the EGFP negative population) (Additional file 3: Figure  3A). In this case the generation of paired DSBs did not enhance the disruption of EGFP, but did enhance concurrent HDR at two proximal sites on the same chromosome. One possible explanation is that individual TALEN pairs generated temporally separated DSB and HDR events, thereby avoiding inducing genomic deletion. This hypothesis is supported by sequence data from the full length PCR products derived from EGFP negative cells, whereby all sequenced events from the two TALEN pairs had an indel corresponding to the TALEN pair A target site whilst the TALEN pair F target site remaining intact. In contrast, when paired guides were used, indels were observed at both target sites in most sequenced events (Additional file 3: Figure  3B). When supplied with a plasmid template spanning the two target sites, paired gRNA/Cas9 and two pairs of TALENs generated similar HDR results whereby few EBFP positive cells were detected in the EGFP positive population (Additional file 3: Figure  3A, Additional file 8: Table 3).

**Fig. 4:**
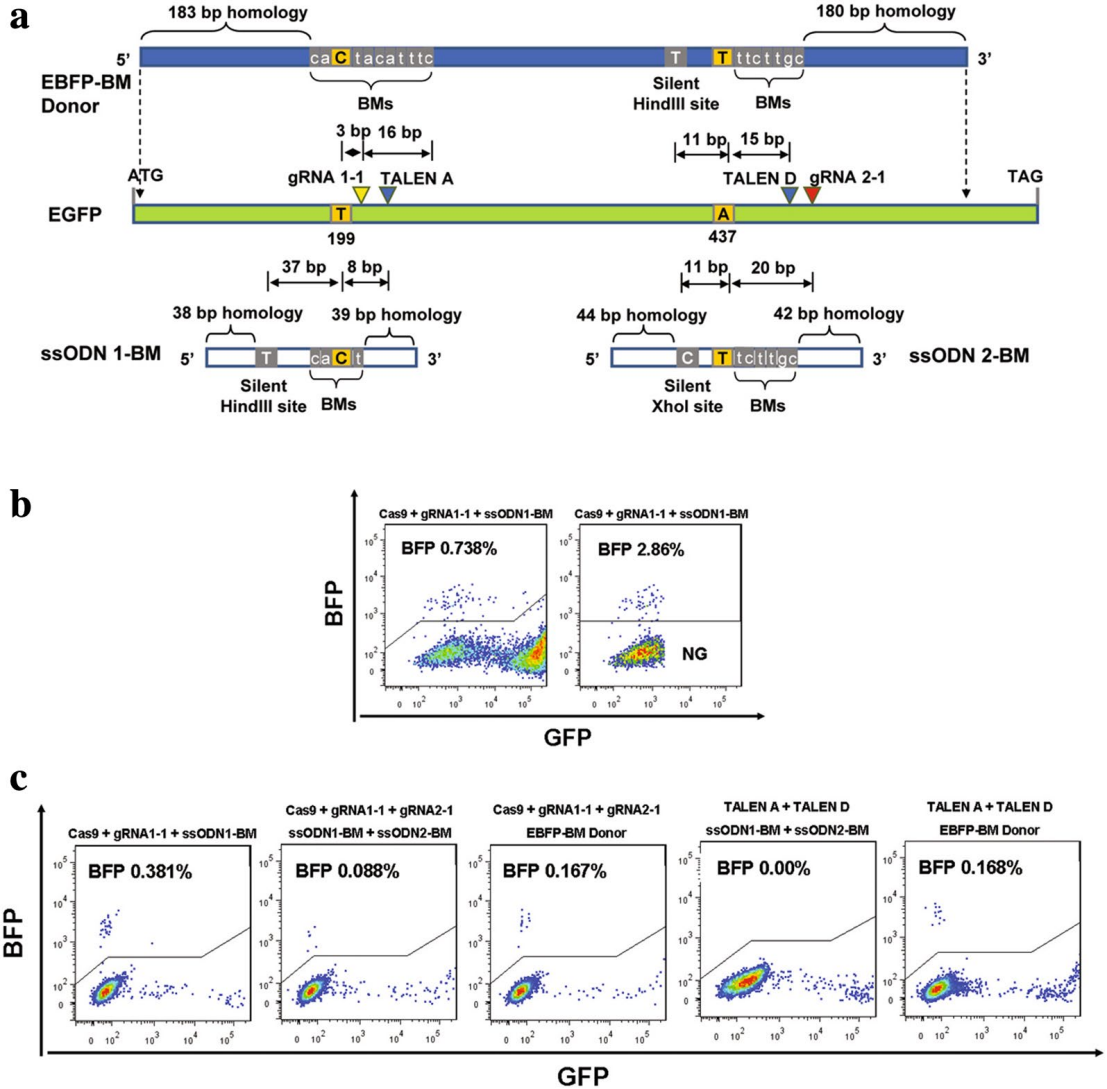
The design of single-stranded oligodeoxynucleotides (ssODNs) and plasmid donor with blocking mutations, and the comparison of HDR efficiencies between TALEN and CRISPR/Cas9. **a** The schematic diagram shows two 120-mer ssODN and a plasmid donor DNA with blocking mutations (BMs) used to incorporate the two substitutions (199T > C) and (437A > T) into the EGFP locus. Predicted cleavage sites of CRISPR/Cas9 or TALEN are indicated by *coloured arrow heads*. A silent G to T mutation was introduced on ssODN1-BM and EBFP-BM plasmid to create a silent HindIII site, and a silent G to C mutation was introduced on ssODN2-BM to create a silent XhoI site. More silent mutations were introduced on ssODN and plasmid template to form the blocking mutations (BMs) for each TALEN arm and gRNA. **b** Representatives of the FACS analysis of EBFP positive cells generated via HDR by using TALEN or CRISPR/Cas9 with donor templates with blocking mutations. NG: EGFP negative cells. **c** The quantification of EBFP positive cells in extended cultured EGFP negative cells sorted from HEK293FT_EGFP_ cells transfected with TALEN or CRISPR/Cas9 conjugated with donor templates with blocking mutations. In each case, a total of 10,000 events were counted

One explanation for the low conversion rate of EGFP to EBFP, when using gRNA/Cas9 or TALEN with either ssODN or plasmid as template for HDR, is that genomic cleavage and NHEJ at the target sites can occur subsequent to any HDR event (i.e. re-cutting). In order to investigate this possibility we designed template molecules (ssODN and plasmid) containing silent blocking mutations (BM) in addition to the desired coding base changes, with the intention that once an HDR event had introduced the desired mutation into the EGFP target sequence, the modified site would differ sufficiently from the original target sequence that re-cutting would not occur (Fig. 4a). The introduction of blocking mutations on oligonucleotide templates substantially improved the HDR efficiency, as evidenced by 0.738 % EBFP positive cells (2.86 % of the EGFP negative population) detected when ssODN1-BM was co-delivered into HEK293FT_EGFP_ cells with gRNA 1-1 and Cas9 (Fig. 4b; Additional file 4: Figure  4A, Additional file 3: Table 3).

Steric hindrance between two adjacent Cas9-gRNA complexes could be a factor that negatively impacts on indel formation. To investigate whether this was likely we co-delivered two guide RNAs (gRNA 1-1 and gRNA 1-2), displaying a 14 bp overlap, concurrently to HEK293FT_EGFP_ cells with Cas9 and ssODN1-BM. Surprisingly, we found that delivery of the pair of guides resulted in significantly more EGFP negative cells than either guide alone; 16.8 % for the pair versus 9.7 % for gRNA 1-1 and 14.5 % for gRNA 1-2. Moreover, paired guides generated a comparable conversion rate to EBFP positive cells (0.66 %) when compared to gRNA 1-1 alone (0.74 %) (Additional file 4: Figure  4A, Additional file 3: Table 3). In contrast, TALEN pair A or C as a single delivery induced less than 0.01 % EBFP conversion with the supply of ssODN1-BM (Additional file 4: Figure  4A, Additional file 3: Table 3).

**Fig. 5:**
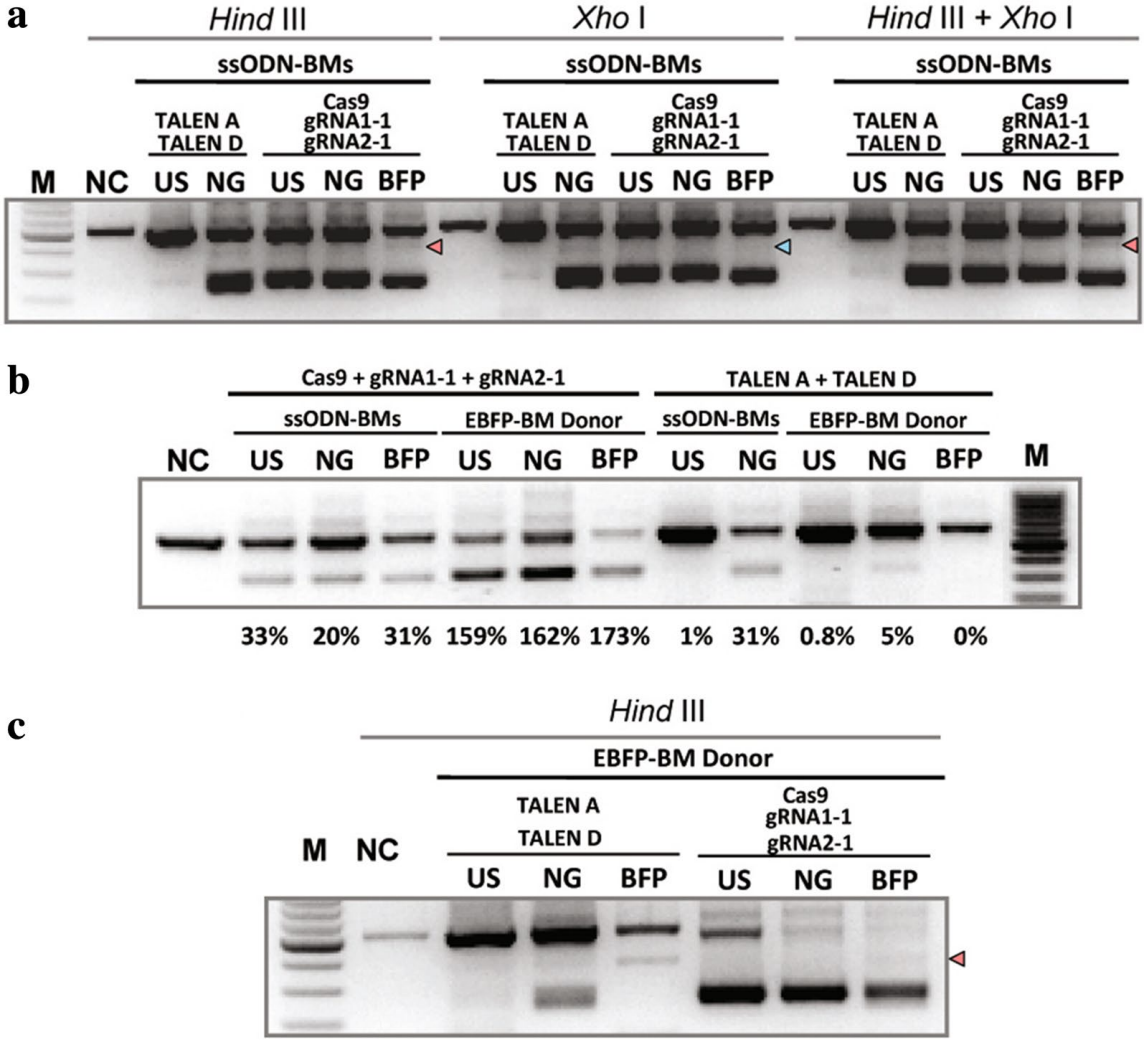
Analysis of HDR mediated by TALEN or CRISPR/Cas9 using RFLP assay. **a** Hind II or Xho -digested, or co-digested genomic DNA isolated from cells derived from HEK293FT_EGFP_ cells transfected with paired Cas9 or two TALEN pairs with ssODN1-BM and ssODN2-BM. NC: genomic DNA from untransfected HEK293FT_EGFP_ cells was used as negative control. US: genomic DNA from unsorted HEK293FT_EGFP_ cells transfect with indicated constructs. NG: genomic DNA from sorted EGFP negative cells. EBFP: genomic DNA from sorted EBFP positive cells from extended cultured EGFP negative populations. *Red* and *green arrow head* indicate the expected size of HindIII-digested and XhoI-digested product respectively. **b** Detection of targeted genomic deletions in differently sorted populations from HEK293FT_EGFP_ cells transfected with indicated constructs. Symbols as shown in (**a**). **c** HindIII-digested genomic DNA isolated from cells derived from HEK293FT_EGFP_ cells transfected with paired Cas9 or two TALEN pairs with EBFP-BM plasmid donor. *Symbols* as those shown in (**a**)

When supplied with both ssODN1-BM and ssODN2-BM, either paired guides/Cas9 or paired TALEN pairs were inefficient at inducing concurrent HDR at both target sites as determined by FACS (Additional file 4: Figure  4B, Additional file 3: Table 3) or RFLP (Fig. 5a). As blocking mutations on the oligonucleotide templates should prevent re-cutting following HDR, HDR at one or both target sites should prevent deletion of the intervening fragment through NHEJ-mediated breakpoint junction re-joining. Sequence analysis of PCR clones derived from sorted EBFP positive cells derived from transfection with gRNA1-1, gRNA2-1, ssODN1-BM, ssODN2-BM, and Cas9, revealed that no concurrent HDR was found from 10 sequenced events (Additional file 5: Figure  5A).

Blocking mutations on the donor plasmid increased the percentage of EBFP positive cells from 0.013 to 0.055 % when gRNA1-1 and gRNA2-1 were co-delivered, with 0.27 % EBFP cells appearing in the EGFP negative population (Additional file 4: Figure  4B, Additional file 8: Table 3). Conversion from EGFP to EBFP positive cells were also detected using other paired guide/Cas9 combinations (Additional file 8: Table 3). Paired delivery of TALEN pairs A and D with the EBFP-BM donor plasmid resulted in a conversion rate of 0.02 % EBFP, or 0.334 % EBFP positive cells in the EGFP negative population (Additional file 4: Figure  4B, Additional file 3: Table 3). In comparison to the simultaneous delivery of 2 ssODNs with blocking mutations, a plasmid with blocking mutations proved a superior donor. The proportion of EBFP positive cells in the EGFP negative population was approximately doubled when plasmid template was utilised instead of the 2 ssODNs (0.167 vs 0.088 %; Fig. 4c). The result was similar when TALEN pairs A and D were co-transfected with the EBFP-BM donor plasmid (0.168 %). By contrast, no EBFP positive cells were detected when ssODN1-BM and ssODN2-BM were used in conjunction with the paired TALENs (Fig. 4c). Sequence analysis of PCR products from EBFP positive cells generated from EBFP-BM mediated HDR by either paired gRNA/Cas9 or two TALEN pairs, showed that simultaneous site conversion events could be induced using either approach (Additional file 5: Figure  5B and C). When supplied with the EBFP-BM plasmid, TALEN pairs were less efficient at inducing genomic deletions than gRNAs, as revealed by PCR (Fig. 5b) but were more efficient at stimulating concurrent HDR as determined by RFLP (Fig. 5c). As Cas9 co-transfected with a ssODN template was more efficient at stimulating HDR at a single target site than simultaneously at adjacent sites (Fig. 4c), we investigated the possibility of carrying out the EGFB to EBFP conversion in a stepwise manner. First we mutated Y145F (437A > T) using the ssODN2-BM in conjunction with either gRNA2-1, gRNA2-2, or both, and subsequently used the same cells to mutate the Y66H (199T > C) using ssODN1-BM and gRNA1-1. Using this approach we generated up to 1.15 % EBFP positive cells as a proportion of the whole population, translating as 4.05 % EBFP positive cells as a proportion of the EGFP negative population (Additional file 4: Figure  4C).

## Discussion

We have carried out a systematic comparison of CRISPR/Cas9 and TALEN editing of an EGFP gene integrated in the genome of HEK293FT cells. Paired gRNAs induced targeted genomic deletion more efficiently than two TALEN pairs, and generated more precisely rejoined breakpoint junctions. When supplied with a plasmid template spanning the paired DSBs, TALENs stimulated homology directed repair (HDR) more efficiently than CRISPR/Cas9, and induced fewer targeted genomic deletions.

Both point mutations and chromosomal deletions can be associated with genetic diseases (Stankiewicz and Lupski 2010). To better understand the roles these genomic modifications play in complex diseases, it would be advantageous to precisely replicate them by targeted modification of higher eukaryotic cells and organisms. Using two DNA editor pairs that are targeted to the same chromosome it is possible to generate deletions of chromosomal segments in cell lines or live organisms (He et al. 2015; Lee et al. 2010; Xiao et al. 2013). It has been well understood that the FokI nuclease of ZFNs and TALENs cleaves DNA within the spacer between the paired binding sites and generates overhangs, which can then be re-joined through NHEJ (Kim et al. 2013b). The NHEJ pathway is extremely flexible and can proceed through many rounds of enzymatic activity involving resection of overhangs by nucleases, gap fill-in by polymerase and DNA ligation. Moreover, when the classical NHEJ pathway is impeded, other cellular repair factors can substitute for it. This substitution, which is called alternative end joining (alt-EJ), backup NHEJ, or microhomology-mediated end joining (MMEJ), varies considerably in efficiency among species and cell types (Lieber and Wilson 2010). Therefore, chromosomal repair by NHEJ can potentially result in diverse products depending on the repair mechanisms that are induced. Heterogeneity associated withmicro-homologies were often observed at the junction of paired ZFN-directed deletion events (Lee et al. 2010) and were also observed at the junction of TALEN-mediated deletion events in this study. In contrast the junctions of paired Cas9 nuclease-directed deletions observed in this study were surprisingly homogeneous, with the breakpoint junctions three or four base pairs upstream of the PAM (Jinek et al. 2012). NHEJ ligation requires the association of two double-strand ends. In one model, the Ku:DNA-PKcs complex forms a tight dimer across the break, such that DNA-PKcs autophosphorylation occurs, at which point protection of the DNA ends is released to allow enzymatic action. In a second model, less rigid associations are achieved by tethering two ends close to each other but leaving the termini free. In this model, with insufficient base pairing of overhangs to hold a double-strand break together, structural components facilitate the equilibria of many potential alignments of the ends with final synapsis (i.e. the holding together of the two DNA ends) achieved only transiently within the active sites of the catalytic enzymes. In addition, micro-homology could be generated de novo even when two blunt ends are joined, which may occur by the compensatory addition or deletion of nucleotides at opposing ends of the double-strand break (Lieber and Wilson 2010). It is noteworthy that this was not observed in the current study. Therefore, the repairing of the blunt ends generated by paired guides/Cas9 by the MMEJ pathway seems an unfavoured outcome. Deletions generated by paired guides/Cas9 could provide an interesting field for further investigation into alternative pathways of NHEJ.

Our comparison of TALENs with CRISPR/Cas9 has shown that the latter is more efficient at generating fragment deletion, and HDR at a single site, but that TALENs plus a plasmid template were more efficient at mediating concurrent HDR at two proximal DSBs with reduced risk of deletion of the intervening chromosomal sequence. If the first step of HDR is limiting, TALENs would be more efficient than CRISPR/Cas9 at initiating the process as they generate overhangs which could be readily used for strand invasion when a dsDNA template spanning the two target sites is present. More efficient initiation of HDR would have the knock-on consequence of reduced end-break rejoining by NHEJ. It is worth noting that ssODNs were less efficient than dsDNA templates at stimulating HDR at concurrent DSBs generated by paired gRNA/Cas9 or paired TALEN pairs (Fig. 5c; Additional file 4: Figure  4B). It is possible that the HDR mediated by ssODNs is through a pathway similar to synthesis-dependent strand annealing (Sung and Klein 2006); instead of invading the homologous dsDNA, the ssODN may anneal directly with the single stand overhang on one side of the DSB and subsequently anneal with the overhang on the other side of the DSB, followed by gap-filling and ligation. When concurrent DSBs occur, the displaced extended single-stranded tail may not find the single-stranded overhang on the other DSB end. Resolution of the exchanged DNA strands can result in crossover, whereby segments of the interacting dsDNA are exchanged. Therefore, TALENs present advantages when stimulating simultaneous gene conversion at two sites within one chromosome when supplied with a dsDNA template spanning the two target sites.

## Conclusions

Through comparison of CRISPR/Cas9 and TALENs on editing an integrated EGFP gene we found that CRISPR/Cas9 was superior for targeted genomic deletion, while TALENs outperformed CRISPR/Cas9 for stimulation of concurrent HDR at proximal sites within a single gene. These two genome editing tools are therefore complementary to each other.

## Additional files

10.1186/s40064-016-2536-3 gRNA expression vectors and donor templates used in this study. (A) U6 promoter based expression scheme for the gRNAs. The use of U6 promoter constrains the first position in the RNA transcript to be a “G”. (B) The target sequences of four gRNAs used in this study are listed. The PAM sequence in red. (C) The sequence of EBFP and EBFP-BM donor templates which were synthesized as gBlocks (IDT). (D) Sequences of oligonucleotide templates used in this study.
10.1186/s40064-016-2536-3 Southern blot analysis of the copy number of the integrated EGFP gene. (A) Schematic diagram of the lenti-virus construct used for generating HEK293FT_EGFP_ cell line. (B) The genomic DNA was digested with NcoI or XhoI, a probe derived from the fragment between EcoRI andNcoI was used for blotting. As EGFP was integrated into genome via the long terminal repeat (LTR)-directed integration, if multiple integration happened, different sized of blotted genomic fragments can be detected.
10.1186/s40064-016-2536-3 A comparison of TALEN and CRIPSR/Cas9 on stimulating HDR with ssODNs or plasmid donor. (A) FACS analysis of EBFP positive cells generated via HDR by using TALEN or CRISPR/Cas9 with indicated donor templates. HEK293FT cell line was used as EGFP and EBFP negative control. HEK293FT_EGFP_ cell line was used as EGFP positive and EBFP negative control. HEK293FT cells transiently transfected with a plasmid encoding EBFP were used as EBFP positive control.NG represents EGFP negative cells. In each case, a total of 10,000 events were counted. (B) Sequence analysis of the editing results on target sites in EGFP negative cells produced by TALEN or CRISPR/Cas9 with ssODN or plasmid templates. Target site PAM sequences in red, and gRNA-matching sequences in bold upper case letters. Target sites of TALEN pair A underlined boldface letters, and highlighted in yellow, and target sites of TALEN pair F highlighted in green. Intended substitution sites were underlined, and highlighted in yellow. Inserted bases are shown in blue. Dashes indicate deleted bases.
10.1186/s40064-016-2536-3 A comparison of TALEN and CRIPSR/Cas9 on stimulating HDR with ssODNs or plasmid donor with blocking mutations. (A) FACS analysis of EBFP positive cells generated via HDR on 199T > C substitution site on EGFP by using TALEN or CRISPR/Cas9 with indicated donor templates. HEK293FT cell line was used as EGFP and EBFP negative control. HEK293FT_EGFP_ cell line was used as EGFP positive and EBFP negative control. HEK293FT cells transiently transfected with a plasmid encoding EBFP were used as EBFP positive control.NG represents EGFP negative cells. In each case, a total of 10,000 events were counted. (B) FACS analysis of EBFP positive cells produced from simultaneous HDR on 199T > C and 437A > T target sites by using two TALEN pairs and paired Cas9 with ssODN-BMs or EBFP-BM templates. (C) FACS analysis of EBFP positive cells produced by sequential HDR on 437A > T and 199T > C target sites by using two paired Cas9 with ssODN-BMs.
10.1186/s40064-016-2536-3 Sequence analysis of cloned PCR products from EBFP positive cells derived from HEK293FT_EGFP_ cells transfected with gRNA1-1, gRNA2-1, Cas9 and ssODN-BMs (A) or EBFP-BM template (B), or TALEN pair A and D with EBFP-BM template (C). Target site PAM sequences in red, and gRNA-matching sequences in bold upper case letters. The target sites of TALEN pair A underlined boldface letters, and highlighted in yellow. The target sites of TALEN pair D highlighted in green. The intended substitution site underlined, and highlighted in yellow. Blocking mutations in red, the silent restriction sites in green and highlighted in yellow, and inserted bases in blue. Dashes indicate deleted bases.
10.1186/s40064-016-2536-3 TALEN sequences.
10.1186/s40064-016-2536-3 Primer sequences.
10.1186/s40064-016-2536-3 Comparison of HDR frequencies between CRIPSR/Cas9 and TALEN using oligonucleotide or plasmid templates.
