# Supplementary figures and images for: Comparison of CRISPR/Cas9 and TALENs on editing an integrated EGFP gene in the genome of HEK293FT cells
Source: Springerplus. 2016 Jun 21;5(1):814. doi: 10.1186/s40064-016-2536-3 (PMC4916124; doi:10.1186/s40064-016-2536-3)

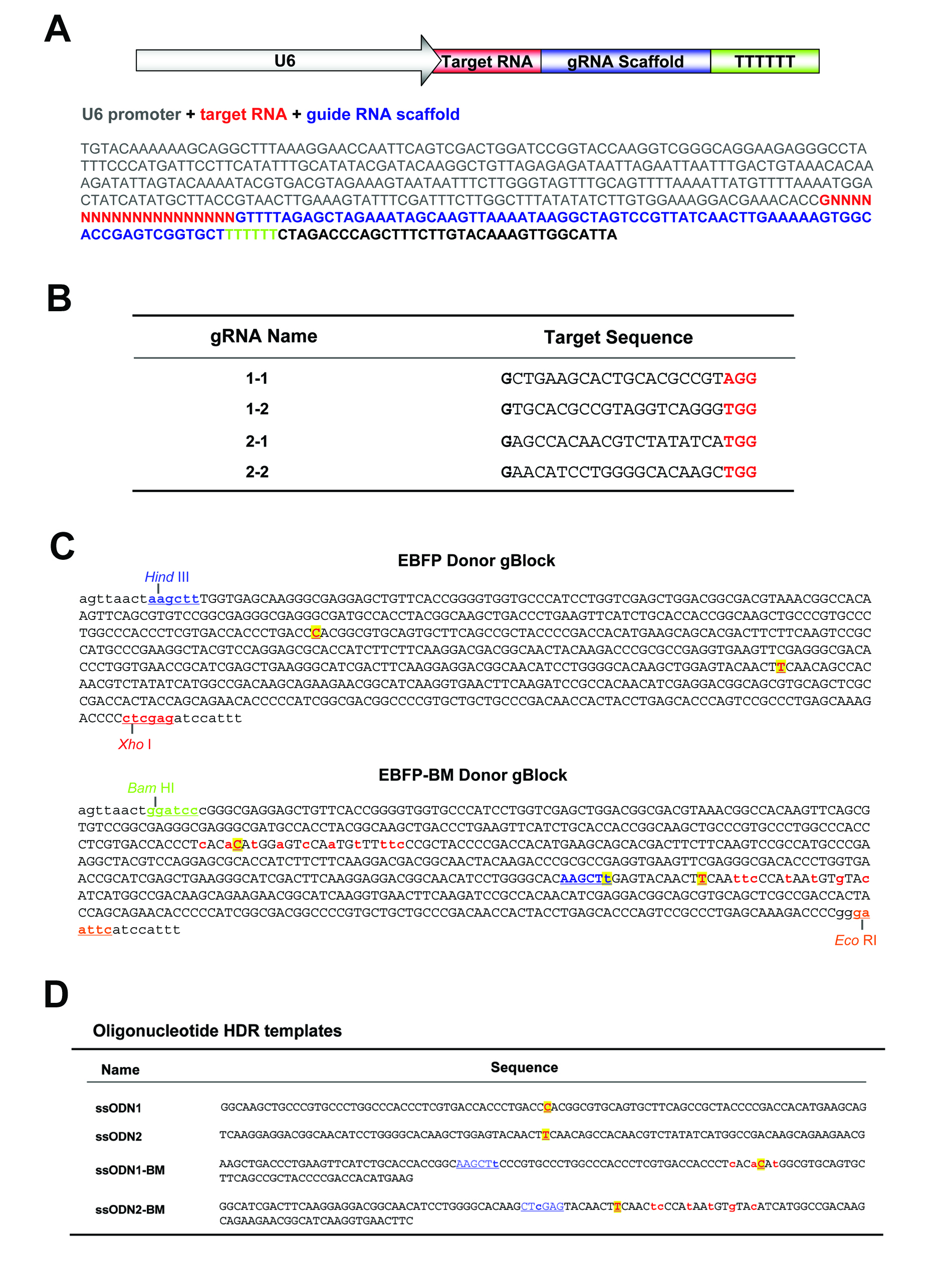

Supplement: Supplementary file 1 — 10.1186/s40064-016-2536-3 gRNA expression vectors and donor templates used in this study. (A) U6 promoter based expression scheme for the gRNAs. The use of U6 promoter constrains the first position in the RNA transcript to be a “G”. (B) The target sequences of four gRNAs used in this study are listed. The PAM sequence in red. (C) The sequence of EBFP and EBFP-BM donor templates which were synthesized as gBlocks (IDT). (D) Sequences of oligonucleotide templates used in this study. [file 40064_2016_2536_MOESM1_ESM.jpg]

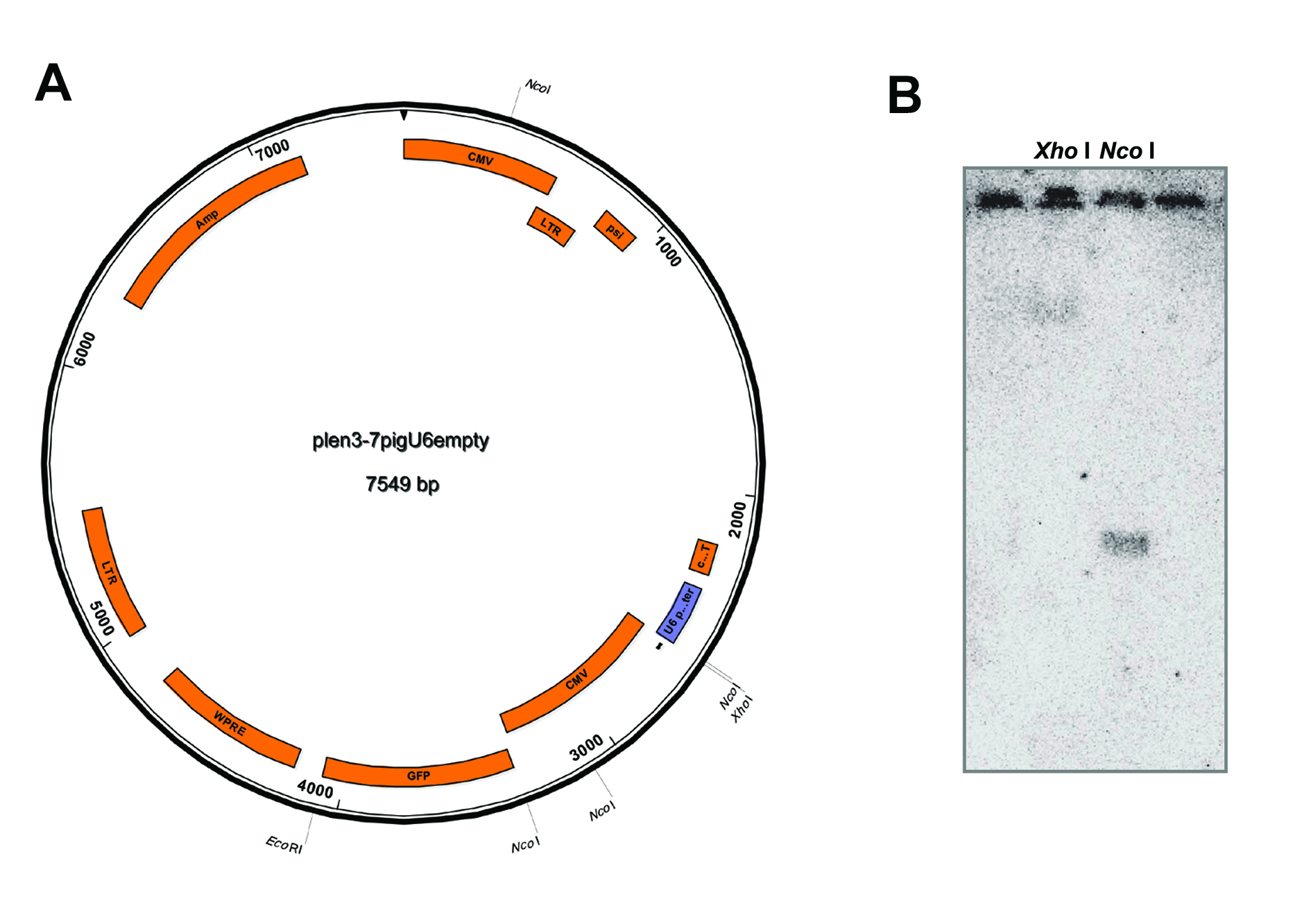

Supplement: Supplementary file 2 — 10.1186/s40064-016-2536-3 Southern blot analysis of the copy number of the integrated EGFP gene. (A) Schematic diagram of the lenti-virus construct used for generating HEK293FTEGFP cell line. (B) The genomic DNA was digested with NcoI or XhoI, a probe derived from the fragment between EcoRI andNcoI was used for blotting. As EGFP was integrated into genome via the long terminal repeat (LTR)-directed integration, if multiple integration happened, different sized of blotted genomic fragments can be detected. [file 40064_2016_2536_MOESM2_ESM.jpg]

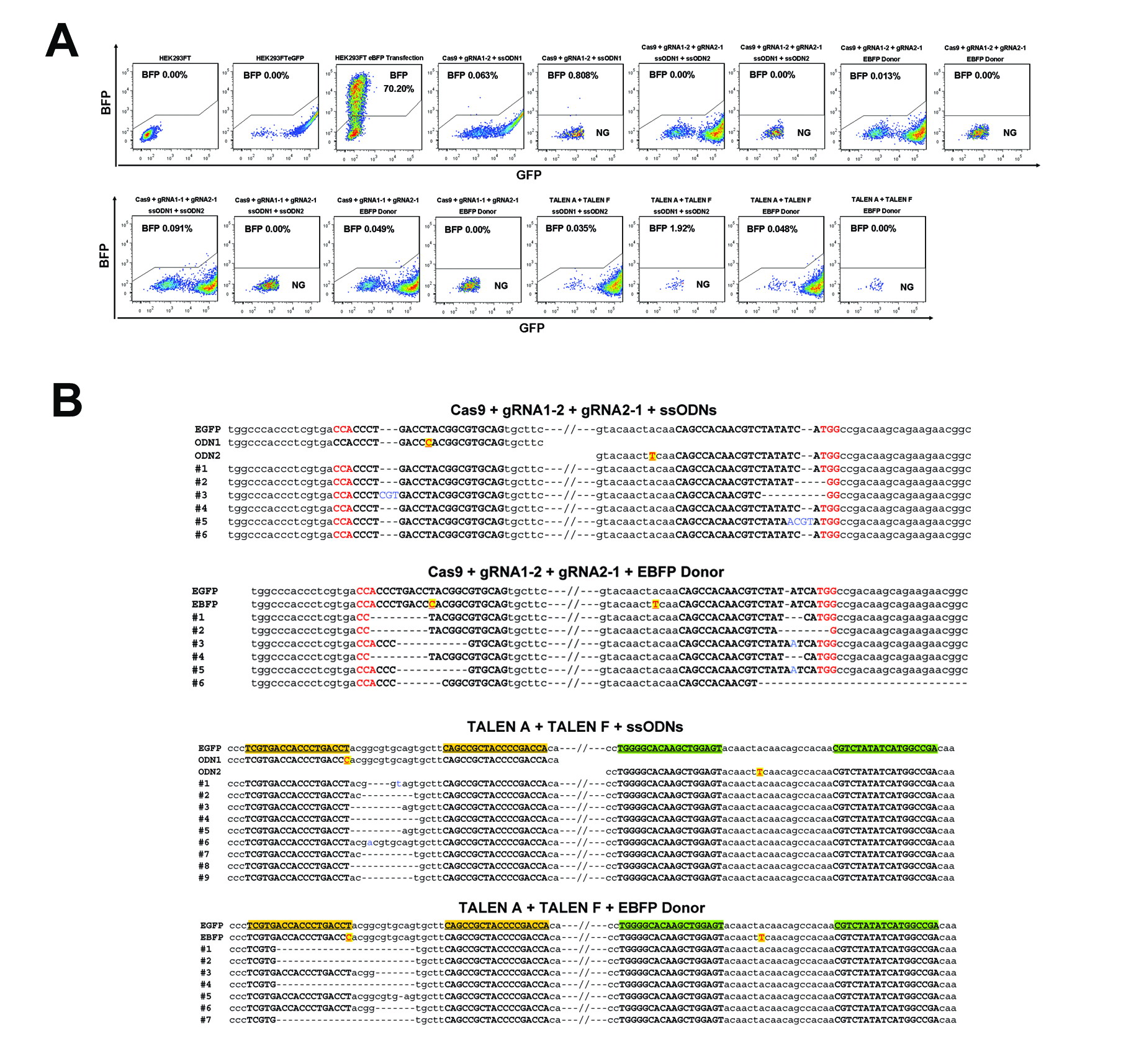

Supplement: Supplementary file 3 — 10.1186/s40064-016-2536-3 A comparison of TALEN and CRIPSR/Cas9 on stimulating HDR with ssODNs or plasmid donor. (A) FACS analysis of EBFP positive cells generated via HDR by using TALEN or CRISPR/Cas9 with indicated donor templates. HEK293FT cell line was used as EGFP and EBFP negative control. HEK293FTEGFP cell line was used as EGFP positive and EBFP negative control. HEK293FT cells transiently transfected with a plasmid encoding EBFP were used as EBFP positive control.NG represents EGFP negative cells. In each case, a total of 10,000 events were counted. (B) Sequence analysis of the editing results on target sites in EGFP negative cells produced by TALEN or CRISPR/Cas9 with ssODN or plasmid templates. Target site PAM sequences in red, and gRNA-matching sequences in bold upper case letters. Target sites of TALEN pair A underlined boldface letters, and highlighted in yellow, and target sites of TALEN pair F highlighted in green. Intended substitution sites were underlined, and highlighted in yellow. Inserted bases are shown in blue. Dashes indicate deleted bases. [file 40064_2016_2536_MOESM3_ESM.jpg]

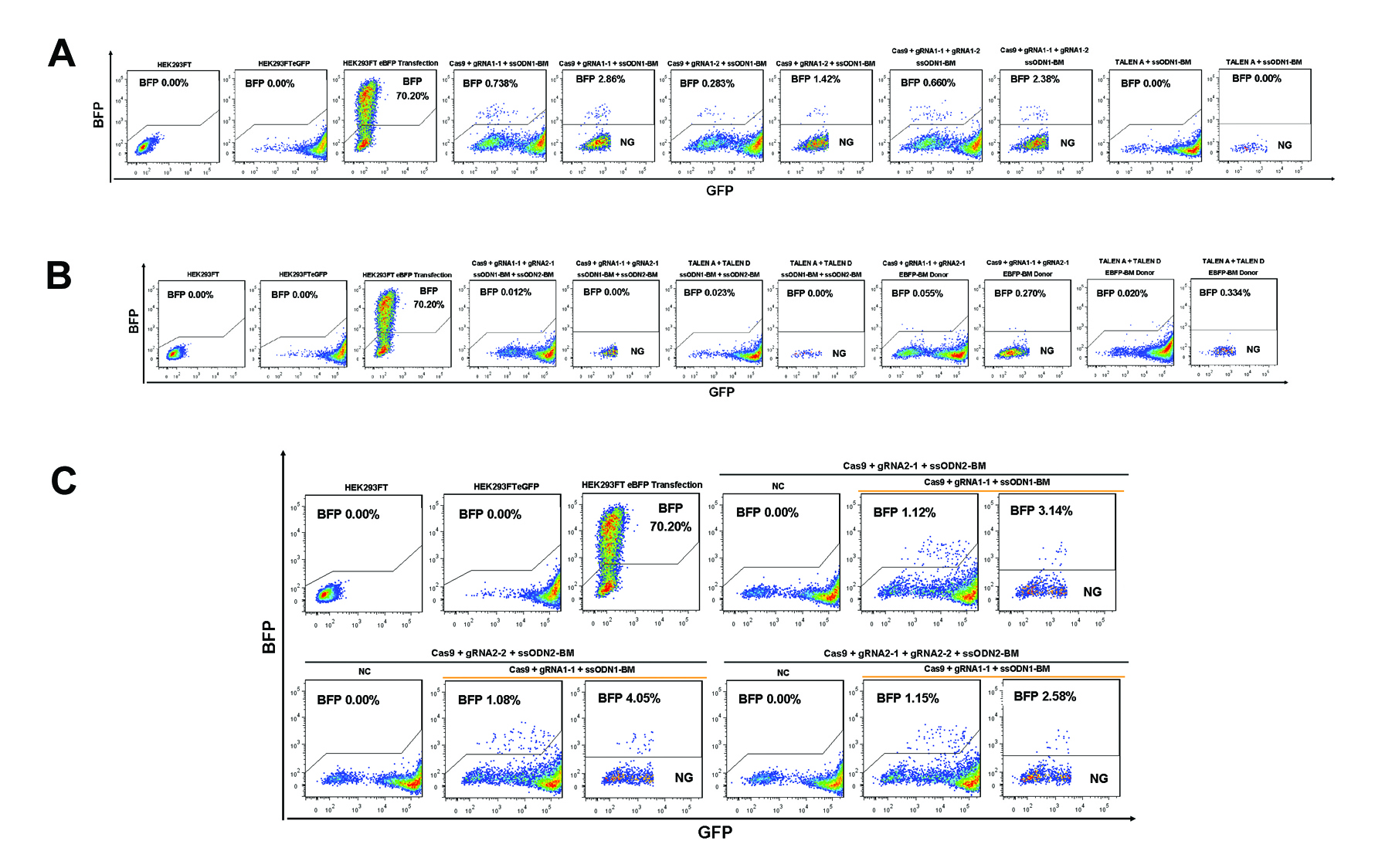

Supplement: Supplementary file 4 — 10.1186/s40064-016-2536-3 A comparison of TALEN and CRIPSR/Cas9 on stimulating HDR with ssODNs or plasmid donor with blocking mutations. (A) FACS analysis of EBFP positive cells generated via HDR on 199T > C substitution site on EGFP by using TALEN or CRISPR/Cas9 with indicated donor templates. HEK293FT cell line was used as EGFP and EBFP negative control. HEK293FTEGFP cell line was used as EGFP positive and EBFP negative control. HEK293FT cells transiently transfected with a plasmid encoding EBFP were used as EBFP positive control.NG represents EGFP negative cells. In each case, a total of 10,000 events were counted. (B) FACS analysis of EBFP positive cells produced from simultaneous HDR on 199T > C and 437A > T target sites by using two TALEN pairs and paired Cas9 with ssODN-BMs or EBFP-BM templates. (C) FACS analysis of EBFP positive cells produced by sequential HDR on 437A > T and 199T > C target sites by using two paired Cas9 with ssODN-BMs. [file 40064_2016_2536_MOESM4_ESM.jpg]

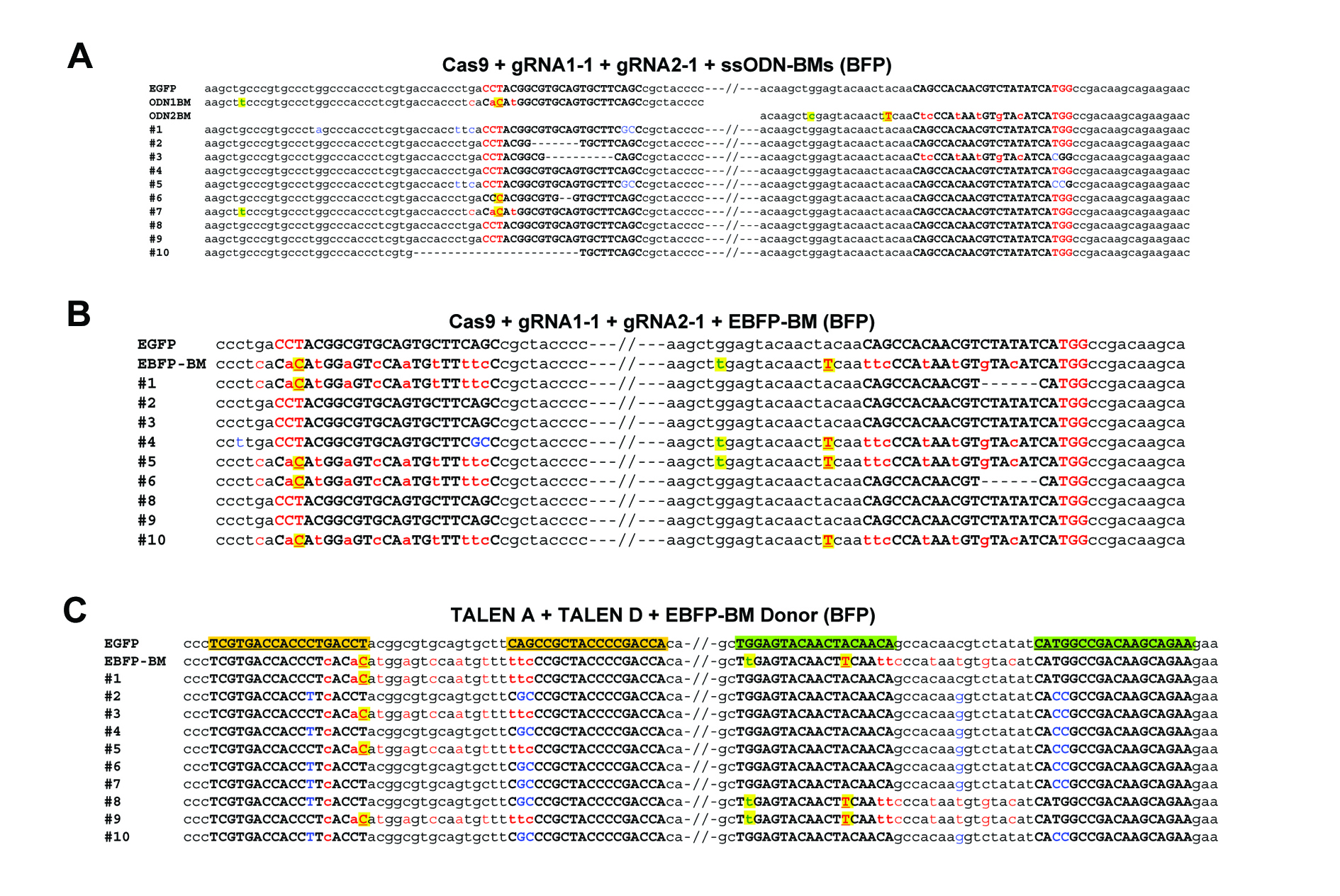

Supplement: Supplementary file 5 — 10.1186/s40064-016-2536-3 Sequence analysis of cloned PCR products from EBFP positive cells derived from HEK293FTEGFP cells transfected with gRNA1-1, gRNA2-1, Cas9 and ssODN-BMs (A) or EBFP-BM template (B), or TALEN pair A and D with EBFP-BM template (C). Target site PAM sequences in red, and gRNA-matching sequences in bold upper case letters. The target sites of TALEN pair A underlined boldface letters, and highlighted in yellow. The target sites of TALEN pair D highlighted in green. The intended substitution site underlined, and highlighted in yellow. Blocking mutations in red, the silent restriction sites in green and highlighted in yellow, and inserted bases in blue. Dashes indicate deleted bases. [file 40064_2016_2536_MOESM5_ESM.jpg]
