# Supplementary material for: Comparison of CRISPR/Cas9 and TALENs on editing an integrated EGFP gene in the genome of HEK293FT cells
Source: Springerplus. 2016 Jun 21;5(1):814. doi: 10.1186/s40064-016-2536-3 (PMC4916124; doi:10.1186/s40064-016-2536-3)
Supplement: Supplementary file 6 — 10.1186/s40064-016-2536-3 TALEN sequences. [file 40064_2016_2536_MOESM6_ESM.doc]

**Supplementary Table 1. TALEN Sequences**

| **TALEN pair** | **TALEN RVD Sequences** | **DNA Target Sequences (Sense Strand)** |
| --- | --- | --- |
| **A** | NG HD NN NG NN NI HD HD NI HD HD HD NG NN NI HD HD NG  NG NN NN NG HD NN NN NN NN NG NI NN HD NN NN HD NG NN | TCGTGACCACCCTGACCTACGGCGTGCAGTGCTTCAGCCGCTACCCCGACCA |
| **C** | NG NN NN HD HD HD NI HD HD HD NG HD NN NG NN NI HD HD  NG HD NN NN NN NN NG NI NN HD NN NN HD NG NN NI NI NN | TGGCCCACCCTCGTGACCACCCTGACCTACGGCGTGCAGTGCTTCAGCCGCTACCCCGA |
| **D** | NG NN NN NI NN NG NI HD NI NI HD NG NI HD NI NI HD NI  NG NG HD NG NN HD NG NG NN NG HD NN NN HD HD NI NG NN | TGGAGTACAACTACAACAATATAGACGTTGTGGCCATGGCCGACAAGCAGAA |
| **F** | NG NN NN NN NN HD NI HD NI NI NN HD NG NN NN NI NN NG  NG HD NN NN HD HD NI NG NN NI NG NI NG NI NN NI HD NN | TGGGGCACAAGCTGGAGTACAACTACAACAGCCACAACGTCTATATCATGGCCGA |
