# Supplementary material for: Comparison of CRISPR/Cas9 and TALENs on editing an integrated EGFP gene in the genome of HEK293FT cells
Source: Springerplus. 2016 Jun 21;5(1):814. doi: 10.1186/s40064-016-2536-3 (PMC4916124; doi:10.1186/s40064-016-2536-3)
Supplement: Supplementary file 7 — 10.1186/s40064-016-2536-3 Primer sequences. [file 40064_2016_2536_MOESM7_ESM.doc]

**Supplementary Table 2.** Primer sequences.

| **Primer Name Sequence (5’ to 3’)** |
| --- |
| Site1 F CTGGACGGCGACGTAAAC  Site2 F GACGTAAACGGCCACAAGTT  Site1 R GGGTGTTCTGCTGGTAGTGG  Site2 R GGGGTGTTCTGCTGGTAGTG  HDR R GAACTCCAGCAGGACCATGT |
