# Supplementary material for: Comparison of CRISPR/Cas9 and TALENs on editing an integrated EGFP gene in the genome of HEK293FT cells
Source: Springerplus. 2016 Jun 21;5(1):814. doi: 10.1186/s40064-016-2536-3 (PMC4916124; doi:10.1186/s40064-016-2536-3)
Supplement: Supplementary file 8 — 10.1186/s40064-016-2536-3 Comparison of HDR frequencies between CRIPSR/Cas9 and TALEN using oligonucleotide or plasmid templates. [file 40064_2016_2536_MOESM8_ESM.doc]

Supplementary Table 3. Comparison of HDR frequencies between CRIPSR/Cas9 and TALEN using oligonucleotide or plasmid templates.

| **Sample** | **NG** | **GFP** | **BFP** | **BFP/NG** |
| --- | --- | --- | --- | --- |
| HEK293FT | 100.00% | 0.00% | 0.00% | 0.00% |
| HEK293FT_eGFP_ | 0.91% | 99.01% | 0.00% | 0.00% |
| HEK293FT eBFP Transfection | 100.00% | 0.00% | 70.20% | 70.20% |
| Cas9 + gRNA 1-2 + ssODN1 | 7.83% | 92.17% | 0.063% | 0.808% |
| Cas9 + gRNA 1-2 + gRNA 2-1 + ssODN1 + ssODN2 | 8.21% | 91.79% | < 0.01% | < 0.01% |
| Cas9 + gRNA 1-1 + gRNA 2-1 + ssODN1 + ssODN2 | 12.20% | 87.80% | 0.091% | < 0.01% |
| TALEN A + TALEN F + ssODN1 + ssODN2 | 0.91% | 99.09% | 0.035% | 1.92% |
| Cas9 + gRNA 1-2 + gRNA 2-1 + EBFP Donor | 10.70% | 89.30% | 0.013% | < 0.01% |
| Cas9 + gRNA 1-1 + gRNA 2-1 + EBFP Donor | 10.30% | 89.70% | 0.049% | < 0.01% |
| TALEN A + TALEN F + EBFP Donor | 0.86% | 99.14% | 0.048% | < 0.01% |
| Cas9 + gRNA 1-1 + ssODN1-BM | 9.70% | 91.30% | 0.738% | 2.86% |
| Cas9 + gRNA 1-2 + ssODN1-BM | 14.50% | 85.50% | 0.283% | 1.42% |
| Cas9 + gRNA 1-1 + gRNA 1-2 + ssODN1-BM | 16.80% | 83.20% | 0.660% | 2.38% |
| TALEN A + ssODN1-BM | 1.46% | 98.54% | < 0.01% | < 0.01% |
| TALEN C + ssODN1-BM | 1.19% | 98.81% | < 0.01% | < 0.01% |
| Cas9 + gRNA 1-1 + gRNA 2-1 + ssODN1-BM + ssODN2-BM | 5.47% | 94.53% | 0.012% | < 0.01% |
| Cas9 + gRNA 1-2 + gRNA 2-1 + ssODN1-BM + ssODN2-BM | 5.12% | 94.88% | < 0.01% | < 0.01% |
| TALEN A + TALEN D + ssODN1-BM + ssODN2-BM | 1.11% | 98.89% | 0.023% | < 0.01% |
| TALEN A + TALEN F + ssODN1-BM + ssODN2-BM | 1.05% | 98.95% | 0.012% | < 0.01% |
| TALEN C + TALEN D + ssODN1-BM + ssODN2-BM | 1.04% | 98.96% | 0.011% | < 0.01% |
| TALEN C + TALEN F + ssODN1-BM + ssODN2-BM | 1.03% | 98.97% | < 0.01% | < 0.01% |
| Cas9 + gRNA 1-1 + gRNA 2-1 + EBFP-BM Donor | 16.10% | 83.90% | 0.055% | 0.270% |
| Cas9 + gRNA 1-1 + gRNA 2-2 + EBFP-BM Donor | 16.90% | 83.10% | 0.021% | 0.063% |
| Cas9 + gRNA 1-2 + gRNA 2-1 + EBFP-BM Donor | 15.90% | 84.10% | 0.011% | 0.067% |
| Cas9 + gRNA 1-2 + gRNA 2-2 + EBFP-BM Donor | 12.40% | 87.60% | 0.032% | 0.086% |
| TALEN A + TALEN D + EBFP-BM Donor | 1.20% | 98.80% | 0.020% | 0.334% |
| TALEN A + TALEN F + EBFP-BM Donor | 1.20% | 98.80% | 0.011% | < 0.01% |
| TALEN C + TALEN D + EBFP-BM Donor | 1.04% | 98.96% | 0.011% | < 0.01% |
| TALEN C + TALEN F + EBFP-BM Donor | 1.52% | 98.48% | 0.010% | < 0.01% |

NG represents GFP negative cells and BFP/NG stands for the percentage of BFP positive cells appeared in the GFP negative population.
